# Length Changes of the Medial Patellofemoral Ligament During In Vivo Knee Motion: An Evaluation Using Dynamic Computed Tomography

**DOI:** 10.1177/03635465231205597

**Published:** 2023-11-13

**Authors:** Miriam R. Boot, Sebastiaan A.W. van de Groes, Hans Dunning, Esther Tanck, Dennis Janssen

**Affiliations:** † Orthopaedic Research Laboratory, Radboud University Medical Center, Nijmegen, the Netherlands; ‡ Department of Orthopaedics, Radboud University Medical Center, Nijmegen, the Netherlands; Investigation performed at Radboud University Medical Center, Nijmegen, the Netherlands

**Keywords:** medial patellofemoral ligament, in vivo, dynamic imaging, knee, patella, computed tomography

## Abstract

**Background:**

Medial patellofemoral ligament (MPFL) reconstruction is associated with high complication rates because of graft overloading from incorrect graft positioning. To improve clinical outcomes, it is crucial to gain a better understanding of MPFL elongation patterns.

**Purpose:**

To assess MPFL length changes in healthy knees from 0° to 90° of dynamic flexion and their relationship with anatomic parameters of the patellofemoral joint.

**Study Design:**

Descriptive laboratory study.

**Methods:**

Dynamic computed tomography scans of an active flexion-extension-flexion movement in 115 knees from 63 healthy participants were evaluated to construct knee joint models. Using these models, the MPFL length was measured as the shortest wrapping path from the Schöttle point on the femur to 3 insertion points on the superomedial border of the patella (proximal, central, and distal). MPFL length changes (%) relative to the length in full extension were calculated, and their correlations with the tibial tuberosity–trochlear groove distance, Caton-Deschamps index, and lateral trochlear inclination were analyzed.

**Results:**

The proximal fiber was the longest in full extension and progressively decreased to a median length of −6.0% at 90° of flexion. The central fiber exhibited the most isometric pattern during knee flexion, showing a median maximal decrease of 2.8% relative to the full extension length and no evident elongation. The distal fiber first slightly decreased in length but increased at deeper flexion angles. The median overall length changes were 4.6, 4.7, and 5.7 mm for the proximal, central, and distal patellar insertion, respectively. These values were either not or very weakly correlated with the tibial tuberosity–trochlear groove distance, Caton-Deschamps index, and lateral trochlear inclination when the anatomic parameters were within the healthy range.

**Conclusion:**

The median MPFL length changed by approximately 5 mm between 0° and 90° of flexion. Proximally, the length continuously decreased, indicating slackening behavior. Distally, the length increased at deeper flexion angles, indicating tightening behavior.

**Clinical Relevance:**

In MPFL reconstruction techniques utilizing the Schöttle point to establish the femoral insertion, one should avoid distal patellar insertion, as it causes elongation of the ligament, which may increase the risk for complications due to overloading.

The medial patellofemoral ligament (MPFL) serves as the primary passive stabilizer that prevents lateral patellar luxation.^6^ With a patellar dislocation, the MPFL nearly always ruptures.^25^ Patellar dislocations are a main concern in midadolescence, with an incidence of 147.7 per 100,000 person-years.^27^ First-time dislocations are usually treated nonoperatively, but despite treatment, over 30% of patients experience a recurrence.^21^ These patients usually undergo MPFL reconstruction with or without additional procedures, depending on the presence of anatomic abnormalities causing the patellar instability, which need correction. Essentially, 3 of these risk factors include trochlear dysplasia, tuberosity lateralization, and patella alta.^7^ During MPFL reconstruction, the ruptured MPFL is replaced with a graft to stabilize the patella and prevent recurrent dislocations. Commonly, the graft is fixed between the Schöttle point at the femur and the superomedial border of the patella, aiming for near-isometric behavior.^1,28^ Yet, around 26% of patients have complications after MPFL reconstruction, including knee pain, restricted range of motion, and recurrent instability, which can ultimately result in osteoarthritis.^10,29,34^ Technical errors, involving incorrect positioning of the femoral or patellar fixation site, have been reported as the primary cause of these complications.^26^ These errors become particularly concerning when they lead to graft tightening during knee flexion, which is associated with worse clinical outcomes.^26^ These complications stress the need for correct graft positioning and elongation patterns.

Knowledge on the physiological strain behavior of the native MPFL is a key step toward reducing the number of complications. To date, there has been little agreement on what the native MPFL length changes are.^14,22^ A recent review by Huber et al^14^ summarized the variability into 1 weighted mean MPFL length change during knee flexion, indicating that, on average, the MPFL exhibits near-isometric behavior over the first 60°, followed by shortening of the ligament with progressive knee flexion. However, the weighted mean was based on relatively small in vivo studies consisting of ≤20 participants and focusing on passive knee flexion. Given the large interstudy variability, further confirmation with a larger sample size is needed. In addition, it is unclear whether these length changes can simply be translated to daily life situations with active knee movements.

Further research is also needed to determine the relationship between MPFL length changes and anatomic parameters of the patellofemoral joint. Cadaveric studies have demonstrated that a greater tibial tuberosity–trochlear groove (TT-TG) distance and patella alta—that is, a greater patellar height relative to the femur—significantly increase MPFL anisometry, showing increased slackening during knee flexion. After reconstruction, this could potentially result in the inability to attain full extension because of excessive graft tension and failure as a consequence.^24,35^ Recently, Tanaka et al^33^ investigated in vivo MPFL length changes from 0° to 50° of flexion in knees with anatomic risk factors for patellar instability, including patella alta, tuberosity lateralization, and trochlear dysplasia. In accordance with cadaveric studies, they showed increased MPFL slackening during knee flexion in the presence of anatomic risk factors, especially when multiple risk factors were present simultaneously.^33^ In practice, patients with anatomic risk factors for patellar instability usually undergo additional procedures to correct their abnormalities, and the optimal surgical treatment method for patellar instability is still a matter of debate.^7^ Therefore, it would be interesting to investigate whether a similar relationship exists between these anatomic parameters and in vivo MPFL length changes in healthy knees.

The present study aimed to assess length changes of the native MPFL from 0° to 90° of dynamic flexion in 100 healthy young participants. The second aim was to quantify the relationship between these length changes and tuberosity lateralization measured using the TT-TG distance, patellar height measured using the Caton-Deschamps index (CDI), and trochlear dysplasia measured using the lateral trochlear inclination (LTI). It was hypothesized that MFPL length change patterns would differ along the width of the MPFL. MPFL length changes were determined using dynamic computed tomography (CT).

## Methods

Institutional review board approval was obtained, and all participants provided written informed consent for secondary use of their data. A data set with static and dynamic CT scans of 100 healthy White participants was used. All participants were aged 18 to 35 years and did not have any history of knee abnormalities or complaints. Participants with functional or congenital disorders and visually diagnosed varus or valgus malalignment were excluded.

### CT Scanning Protocol

All participants underwent static and dynamic CT of their knee joints between 2020 and 2021, as previously described in detail.^9^ First, a high-resolution static CT scan (Aquilion ONE CT scanner; Canon Medical Systems) of both knee joints was obtained with the participant in the supine position (field of view: 50 cm; mean voxel size: 0.71 × 0.71 × 0.80 mm). Subsequently, participants were positioned in a semiseated posture at the end of the scanner table with their legs in 90° of flexion, after which a dynamic CT scan was obtained of both knee joints (field of view: 16 cm; mean voxel size: 0.98 × 0.98 × 0.50 mm). During dynamic CT, participants actively extended their legs to full extension and flexed back to 90° of flexion in 10 seconds. Before scanning, the movement was practiced. A sequence of 41 medium-resolution CT scans was taken of the knee joint at different flexion angles.

### Construction of 3-Dimensional Bone Models

After acquisition, all CT scans were subjected to further processing. First, the femur, patella, and tibia of both legs were automatically segmented using a convolutional neural network (Dice scores; femur: 0.99; patella: 0.96; tibia: 0.98).^20^ The generated masks were then converted to 3-dimensional (3D) surface meshes and optimized (automatically remeshed and smoothed) using MATLAB (Version R2021b; MathWorks). Afterward, the static CT meshes of the femur, patella, and tibia were registered to each of the dynamic CT meshes with point set registration using a coherent point drift algorithm, followed by intensity-based B-spline rigid registration using the elastix toolbox.^19^ This allowed combining anatomic details of the static CT scans with kinematic data of the dynamic CT scans. Registrations were manually checked and interpolated for every degree of knee flexion to facilitate data comparison between participants. The accuracy of the registrations was approximately 1° and 1 mm for rotation and translation, respectively.^9^

### Exclusion Criteria and Knee Measurements

**Figure 1.**
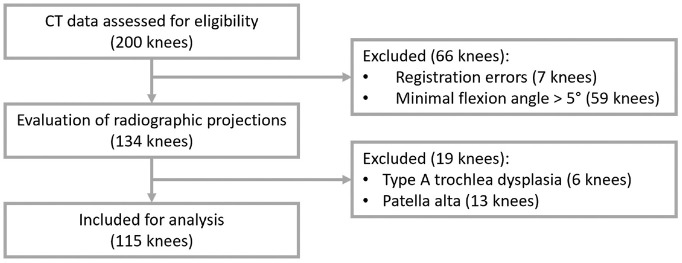
Flowchart of participant inclusion. CT, computed tomography.

Of the 200 knees assessed for eligibility, 85 knees were excluded (Figure 1). There were 7 knees excluded because of registration errors caused by significant image artifacts or incorrect positioning in the scanner, leading to the loss of positional information of either of the bones. An additional 59 knees were excluded, as they did not reach a flexion angle ≤5°, preventing an assessment of the MPFL length in (near-)full extension. These participants either did not fully extend their legs during dynamic CT, despite the fact that the flexion-extension-flexion movement had been practiced, or experienced difficulties with full knee extension in this open-kinetic chain seated setup without back support. The remaining 134 knees were evaluated for the presence of trochlear dysplasia, patella alta or baja, or an abnormal TT-TG distance to exclude those with abnormal anatomic parameters of the patellofemoral joint. To perform these measurements, we simulated lateral radiographic images using the static CT scans in extension and dynamic CT scans at the flexion angle closest to 30°. Subsequently, the simulated radiographic images were evaluated by an experienced orthopaedic surgeon (S.A.W.vdG.) specializing in knee surgery (Appendix Figures A1 and A2, available in the online version of this article). On the basis of this evaluation, 19 knees were excluded because of low-grade type A dysplasia according to the criteria of Dejour et al^8^ or patella alta measured as a CDI >1.2 in either of the knees forming a pair. The TT-TG distance was derived automatically from the CT scans to assess the lateral insertion of the patellar tendon^8^ and remained below the accepted surgical threshold of 20 mm. The LTI was assessed as a quantitative measure of trochlear morphology. The LTI was obtained in the most proximal part of the trochlear groove, where the notch appeared as a Roman arch. The LTI was defined as the angle between the lateral ridge of the trochlear groove and the posterior condylar axis.^4^ The remaining 115 knees of 63 participants (15 men, 48 women) were included for final analysis (Table 1).

**Table 1:** Participant and Knee Characteristics^*a*^

	Value
Participant characteristics (N = 63)	
Age, y	24.6 ± 3.4 (19-34)
Male:female sex, n	15:48
Length, cm	172.9 ± 8.4 (158-198)
Weight, kg	67.4 ± 10.1 (54-98)
Body mass index	22.5 ± 2.7 (17.7-34.3)
Knee characteristics (N = 115)	
Right:left side, n	61:54 (52 pairs)
CDI	1.0 (0.9-1.1) [0.7-1.2]
TT-TG distance, mm	12.3 (10.2-14.2) [0.2-19.4]
LTI, deg	20.0 (18.0-22.8) [11.4-28.6]

a Data are shown as mean ± SD (range) or median (interquartile range) [range] unless otherwise indicated. CDI, Caton-Deschamps index; LTI, lateral trochlear inclination; TT-TG, tibial tuberosity–trochlear groove.

### Patellofemoral Attachments

**Figure 2.**
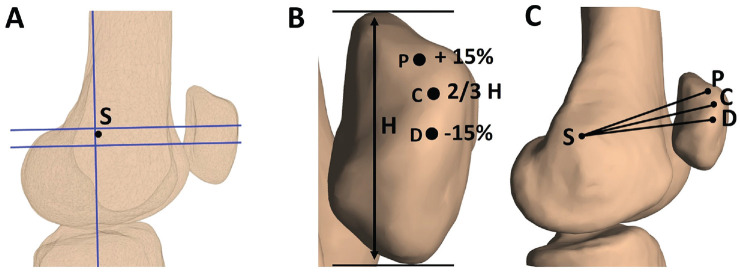
Lateral views of a left knee in extension showing medial patellofemoral ligament (MPFL) attachments. (A) The Schöttle point (*S*) was selected as the femoral origin. To do so, 3-dimensional (3D) knee models were converted to 2-dimensional lateral representations. The Schöttle point was located 1 mm anterior to the posterior cortex extension line (vertical line) and 2.5 mm distal to a perpendicular line that intersected the origin of the medial femoral condyle (upper horizontal line). For illustration purposes, the Blumensaat line is shown as well (lower horizontal line). (B) Overall, 3 points were selected as the patellar insertions. The central insertion (*C*) was selected at the proximal one-third of the total patellar height (*H*). The proximal (*P*) and distal (*D*) insertions were selected at 15% above and below the central insertion along the medial patellar border, respectively. (C) MPFL attachment points on a 3D bone surface model.

The femoral and patellar attachment sites that were selected correspond with anatomic attachments frequently described in the literature.^1^ The Schöttle point is commonly regarded as the radiographic femoral origin of the MPFL.^28^ To determine the Schöttle point, we established the anatomic femoral coordinate system of each static femoral surface model, as previously described by Chen et al.^5^ Subsequently, the femoral surface models were rotated to the mediolateral view that superimposed the femoral condyles. Afterward, the models were converted to a 2-dimensional representation. The Schöttle point was identified based on the posterior extension line and a perpendicular line that intersected the origin of the medial femoral condyle (Figure 2A).^28^ The 2-dimensional Schöttle point was then projected onto the 3D femoral model to obtain the 3D coordinates of the femoral insertion (Figure 2C). The patellar insertion of the MPFL is wider than the femoral origin and spans along the proximal medial border.^1^ Therefore, 3 points were selected to describe the patellar insertion: a central insertion at the proximal one-third of the patellar height and a proximal insertion and a distal insertion 15% above and below the central insertion, respectively (Figure 2, B and C). The area covered by these points represented the normal patellar insertion width according to a systematic review by Aframian et al.^1^ Also, the proximal one-third of the patellar height has generally been considered as a suitable attachment site for single-bundle MPFL reconstruction.^36^

**Figure 3.**
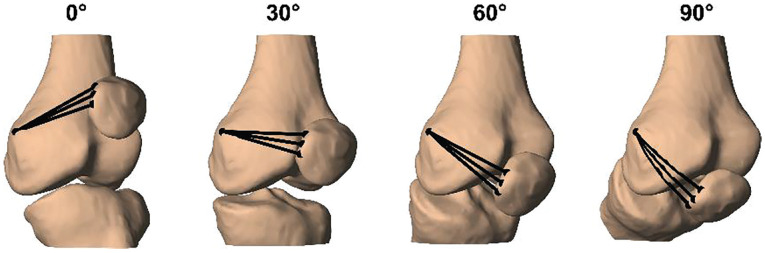
Medial patellofemoral ligament (MPFL) length changes for a left knee from 0° to 90° of knee flexion. The shortest wrapping path running over the femoral condyle was selected as the MPFL length. Wrapping is visible by the curved path of the MPFL fibers at 90° of flexion.

Then, 3 virtual MPFL fibers were created between the femoral and patellar attachment points. To simulate wrapping of the MPFL around the femur, we projected direct lines connecting the femoral and patellar attachment sites onto the bony surface of the femoral condyle using a convex hull algorithm. Subsequently, the shortest wrapping path of each MPFL fiber was determined by rotating the femur around a straight line connecting the attachment point.^2^ This allowed selecting the path with the least resistance as the MPFL length for each flexion angle (Figure 3). Finally, MPFL length changes were assessed from 0° to 90° of flexion.

### Statistical Analysis

The primary outcome was the change in MPFL length during knee flexion. The absolute MPFL length changes in all 115 knees were converted to MPFL length changes (%) relative to the length in full extension. This way, the length changes were not affected by initial differences in MPFL length as a result of anatomic variation. A decrease in MPFL length relative to the full extension length indicated MPFL slackness, and an increase indicated MPFL tightening. MPFL length changes were similar during knee flexion and knee extension. However, only a minor fraction of the participants reached a knee angle of 90° within the scanning time in the flexion phase, as most participants performed the flexion-extension-flexion movement too slowly (Appendix Figure A3, available online). Therefore, the extension phase was used to describe MPFL length changes in this article. For clarity, the MPFL length changes (%) were presented from 0° to 90° rather than from 90° to 0° of flexion. The overall length change was defined as the absolute difference between the maximal and minimal MPFL length between 0° and 90° of flexion. Normality was established using the Shapiro-Wilk test. The MPFL length changes (%) were presented as the median (interquartile range [IQR]), as the data did not meet the assumption of normality for all flexion angles. Spearman rank correlation coefficients were calculated to examine correlations between the MPFL length and length changes and anatomic parameters of the patellofemoral joint, including the TT-TG distance, CDI, and LTI. Differences in MPFL length changes (%) between the 3 fibers were analyzed per flexion angle using the Friedman test. The Tukey post hoc test was performed in case of significant differences. Statistical analyses were performed using MATLAB, with statistical significance set at *P* < .05.

## Results

### MPFL Length Change Patterns

**Figure 4.**
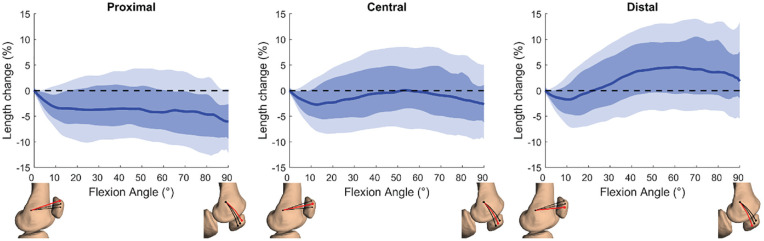
Medial patellofemoral ligament (MPFL) length changes (%) relative to full extension between 0° and 90° of knee flexion for the proximal, central, and distal patellar attachments. Solid lines represent interparticipant medians, dark shading represents the interquartile range (IQR), and light shading represents ±1.5 IQR (12.5%-87.5%). The horizontal dotted line represents the isometric behavior of the MPFL. Not all participants reached a flexion angle of 90° within the scanning time. The flexion angles represent MPFL length changes from a proportion of the participants (37% reached a flexion angle of 90°, 80% reached a flexion angle of 80°, and 97% reached a flexion angle of 70°).

The median length of the proximal fiber was the longest in full extension and progressively decreased at deeper flexion angles to −6.0% (IQR, –9.4% to −2.6%) at 90° of flexion (Figure 4). The central fiber exhibited the most isometric pattern during knee flexion; the median fiber length decreased to 2.8% during the first degrees of flexion, was restored to the full extension length at 50° of flexion, and subsequently decreased to −2.7% (IQR, –6.2% to 1.1%) at 90° of flexion. The median length of the distal fiber initially decreased to –1.8% during the first 10° of flexion and increased at deeper flexion angles to 4.6% (IQR, –0.7% to 9.4%) maximally at 60° of flexion. Substantial variation in MPFL length changes (%) was observed between participants for all 3 MPFL fibers, as shown by the shading in Figure 4. The observed length changes were significantly different between the 3 fibers at all angles from 1° to 90° of flexion (*P* < .05).

### Correlation Between MPFL Length and Anatomic Parameters

The mean MPFL length in full extension was 58.3 ± 5.0 mm for the proximal patellar fiber, 55.7 ± 4.9 mm for the central patellar fiber, and 53.6 ± 4.8 mm for the distal patellar fiber. There was a weak positive correlation between the MPFL length in full extension and the CDI (*r*_s_ = 0.25-0.29) and no significant correlation with the TT-TG distance or LTI (Table 2). The median overall length changes from 0° to 90° of flexion were 4.6 mm (IQR, 3.2-6.0 mm), 4.7 mm (IQR, 3.3-6.0 mm), and 5.7 mm (IQR, 3.6-8.1 mm) for the proximal, central, and distal patellar insertions, respectively. A weak positive correlation was observed between the (absolute and relative) proximal fiber length change and the TT-TG distance (absolute: *r*_s_ = 0.24; relative: *r*_s_ = 0.26) as well as between the distal fiber length change and the LTI (absolute: *r*_s_ = 0.23; relative: *r*_s_ = 0.23) (Table 2). No correlation was detected between the proximal fiber length change and the CDI or LTI; between the central fiber length change and the CDI, TT-TG distance, or LTI; and between the distal fiber length change and the CDI or TT-TG distance.

**Table 2:** Spearman Correlation (*r*_s_) and Statistical Significance (*P*) Between MPFL Length and Length Changes and Anatomic Parameters of Patellofemoral Joint^*a*^

	MPFL Length, mm			Overall MPFL Length Change, mm			Overall MPFL Length Change Relative to Maximal Length, %		
	Proximal	Central	Distal	Proximal	Central	Distal	Proximal	Central	Distal
CDI	*r*_s_ = 0.29	*r*_s_ = 0.27	*r*_s_ = 0.25	*r*_s_ = 0.15	*r*_s_ = 0.14	*r*_s_ = 0.10	*r*_s_ = 0.13	*r*_s_ = 0.12	*r*_s_ = 0.08
	***P* = .002**	***P* = .003**	***P* = .007**	*P* = .11	*P* = .13	*P* = .29	*P* = .15	*P* = .19	*P* = .40
TT-TG distance, mm	*r*_s_ = −0.03	*r*_s_ = −0.06	*r*_s_ = −0.06	*r*_s_ = 0.24	*r*_s_ = 0.07	*r*_s_ = −0.01	*r*_s_ = 0.26	*r*_s_ = 0.11	*r*_s_ = 0.02
	*P* = .77	*P* = .54	*P* = .51	***P* = .008**	*P* = .47	*P* = .89	***P* = .005**	*P* = .24	*P* = .81
LTI, deg	*r*_s_ = −0.09	*r*_s_ = −0.12	*r*_s_ = −0.14	*r*_s_ = −0.17	*r*_s_ = 0.15	*r*_s_ = 0.23	*r*_s_ = −0.18	*r*_s_ = 0.15	*r*_s_ = 0.23
	*P* = .33	*P* = .20	*P* = .15	*P* = .06	*P* = .12	***P* = .01**	*P* = .06	*P* = .10	***P* = .02**

a Significant *P* values are indicated in bold format. CDI, Caton-Deschamps index; LTI, lateral trochlear inclination; MPFL, medial patellofemoral ligament; TT-TG, tibial tuberosity–trochlear groove. See Appendix Figure A4 (available online) for scatterplots illustrating the relationship between the overall MPFL length change and the CDI, TT-TG distance, and LTI.

## Discussion

The most important findings of this study were that the MPFL is an anisometric ligament with a median length change of around 5 mm between 0° and 90° of flexion. MPFL length changes depended on the patellar attachment site, confirming our hypothesis. Proximally, there was a continuous decrease of the median length from 0° to 90° of flexion, and distally, there was an increase of the median length for flexion angles >11°. The central MPFL fiber exhibited the most isometric behavior, with a median decrease in length of 2.8% maximally relative to the full extension length and no evident elongation. The absolute MPFL length in extension and overall MPFL length changes did not correlate or weakly correlated with the TT-TG distance, CDI, and LTI, which were within the healthy range in the present study.

Several studies have focused on MPFL length changes during knee flexion, demonstrating great variability.^11,12,18,30-32,37^ Recently, Huber et al^14^ determined the weighted mean MPFL length change pattern based on in vivo studies, mostly corresponding with the central median MPFL length change observed in our study. In agreement with their study, we found a decrease in MPFL length at flexion angles >60°. Also, the MPFL length changes that we observed were in the same order of magnitude (~5 mm). In contrast to their findings, however, we observed a decrease in MPFL length during early flexion, whereas they suggested an isometric behavior up to 60°. These differences may be related to the variability in the methodological setup. Previous studies have mostly measured MPFL length changes at 0°, 30°, 60°, 90°, and 120° of flexion and, therefore, might not have captured the decrease in MPFL length during early flexion.^13,15,31,37^ The discrepancies might also be related to differences in muscle activity. We assessed MPFL length changes during an active flexion-extension-flexion movement, whereas the weighted mean MPFL length change was based on studies assessing MPFL length changes with passive knee flexion. Previous research has demonstrated that active knee extension from 30° to full extension significantly increases lateral patellar translation and lateral tilting compared with passive incremental knee extension.^3^ Hence, muscle contraction presumably also affects the observed MPFL length changes. Next to muscle contraction, weightbearing also has an effect on patellofemoral kinematics.^16,17,23^ Yet, our length change patterns largely correspond with a recent study investigating MPFL length changes during a weightbearing lunge motion, also demonstrating a decrease in MPFL length during early flexion.^18^ This finding may conceivably indicate that the importance of active weightbearing relative to active nonweightbearing knee motion may be secondary for the assessment of MPFL length changes compared with interindividual variability.

In accordance with previous findings, we observed a dependency of the MPFL length on the patellar attachment site, with a decrease in median length during the entire flexion movement proximally and an increase in median length at deeper flexion angles distally.^18^ This finding indicates that a distal patellar insertion, that is, half the total patellar height, should be avoided in MPFL reconstruction techniques utilizing the Schöttle point to establish the femoral insertion, as the increase in length may cause overloading, resulting in complications.^26^ Yet, a distal patellar insertion is not expected to be problematic for all patients, as some knees show no or a minimal increase in MPFL length during knee flexion.

Cadaveric studies have shown that patella alta and a TT-TG distance >20 mm increase MPFL anisometry during knee flexion.^24,35^ Recently, Tanaka et al^33^ demonstrated a similar relationship in vivo in asymptomatic knees with anatomic risk factors for patellar instability, including patella alta, tuberosity lateralization, and trochlear dysplasia. In our study with healthy participants, we did not find any profound effects of the TT-TG distance, CDI, or LTI on MPFL length changes, as these were in the normal range (CDI <1.2; TT-TG distance <20 mm; LTI <11°^4^). This result could indicate that when the TT-TG distance, CDI, and LTI are within the healthy range, a low risk of graft failure due to excessive anisometry and tension might be expected if a distal patellar insertion is avoided.

Finally, our results have implications for the flexion angle at which the graft is tensioned during reconstruction. It is generally accepted that the MPFL should be reconstructed at the flexion angle with the maximal graft length.^14^ To date, however, there is controversy about the flexion angle at which the MPFL is at maximal tension. Full extension,^18^ 30°,^37^ and 30° to 60°^14^ of knee flexion have all been suggested as suitable. The results of this study indicate that graft fixation between 40° and 60° of flexion, together with a central patellar insertion, that is, at the proximal one-third of the total patellar height, may be favorable for most patients, as the MPFL is subjected to the highest strain at these flexion angles. Although our results suggest that graft fixation in full extension could be an option, in practice, fixation in full extension would be challenging because of the lack of proper guidance of the patella by the trochlear groove. Consequently, surgeons may encounter difficulties in selecting an appropriate graft tension.

A major strength of this study was its methodology and large sample size. Because of the combination of dynamic and static CT, we were able to generate models of active knee motions with high spatial and temporal resolution. Additionally, the dynamic CT scans allowed the creation of radiological images to evaluate the patellofemoral joint anatomy with anatomic parameters. MPFL length changes were extracted fully automatically, which allowed the inclusion of a large sample size of 115 healthy knees. Together, this enabled identifying MPFL length changes in a consistent manner.

Some limitations should be considered. A major limitation is the lack of knowledge on participant-specific MPFL attachment sites. In all knees, we defined MPFL attachment sites at equal locations, whose selection was based on anatomic studies. Therefore, the utilized and native MPFL attachment sites might differ, resulting in MPFL length changes that did not fully represent clinical practice. Nevertheless, it was the best possible method to assess native MPFL length changes, as CT hampered the assessment of participant-specific attachment sites and it resembled the situation after reconstruction of the MPFL. An additional limitation is the absence of soft tissue in the knee models, as the assessment was impeded by the CT scans. Therefore, we were unable to exactly determine the actual path of the MPFL. Instead, we defined MPFL length changes as the shortest distance between the attachment sites over the bony surface of the femoral condyle. This approach may have led to a mild underestimation of the native MPFL length. Another limitation relates to the assessment of the CDI and trochlear dysplasia. As radiographs were lacking, this study converted CT scans to lateral radiographic images, which were of lower quality. Also, the CDI and trochlear dysplasia were measured by only 1 orthopaedic surgeon specializing in knee surgery (S.A.W.vdG.). Both limitations may have introduced inaccuracies. However, the assessment of these measures using simulated radiographic images best corresponded with assessments in clinical practice. Moreover, our approach allowed the evaluation of true lateral projections with (near-)perfect superimposition of the femoral condyles, which is challenging in clinical practice. This approach prevented potential inaccuracies in anatomic parameters associated with rotational errors of simulated radiographic images. Also, the characteristics of the healthy participants in this study do not fully correspond with the “average” patient undergoing MPFL reconstruction because of patellar dislocations. First, there was a sex imbalance, as our study population consisted of 3 times more women than men. In practice, women account for 60% of the patients undergoing MPFL reconstruction.^29^ Second, our study population was limited to White participants, and it is uncertain whether similar MPFL length change patterns can be expected in another population. However, the mean age of the participants in our population was 24.6 years, which to a great extent agrees with the mean patient age associated with MPFL reconstruction of approximately 24 years.^29^ Furthermore, the proximal femur and distal tibia were not included in the CT scans, as the field of view of CT scans was limited to the knee joint. Therefore, quantitative analysis of femoral and tibial torsional deformities and valgus malalignment could not be performed. This limitation was partly overcome by the exclusion of knees with varus and valgus malalignment based on a visual diagnosis. Also, not including the hip region on CT allowed us to keep the effective radiation dose low. Last, MPFL length changes were assessed during a nonweightbearing open-kinetic chain active flexion-extension-flexion movement, which might not be representative of different activities during daily life.^17^ However, our approach is more realistic than that of previous studies examining changes in MPFL length during passive knee flexion. Notwithstanding these limitations, this study offers valuable insights into length changes of the native MPFL during knee flexion, which is essential to obtain a better understanding of MPFL reconstruction and its related complications. Future research should focus on the assessment of MPFL length changes during different loading conditions, especially during demanding activities when the MPFL is under tension and prevents excessive lateral patellar translation.

## Conclusion

The MPFL had a median overall length change of around 5 mm between 0° and 90° of flexion. Length changes varied depending on the patellar attachment site. With a proximal attachment site, the median length decreased from 0° to 90° of flexion. A distal attachment site led to an increase of the median length at deeper flexion angles. No relevant effect of the TT-TG distance, CDI, or LTI on the overall length changes of the MPFL was observed.

## Supplemental Material

sj-pdf-1-ajs-10.1177_03635465231205597 – Supplemental material for Length Changes of the Medial Patellofemoral Ligament During In Vivo Knee Motion: An Evaluation Using Dynamic Computed Tomography Click here for additional data file. Supplemental material, sj-pdf-1-ajs-10.1177_03635465231205597 for Length Changes of the Medial Patellofemoral Ligament During In Vivo Knee Motion: An Evaluation Using Dynamic Computed Tomography by Miriam R. Boot, Sebastiaan A.W. van de Groes, Hans Dunning, Esther Tanck and Dennis Janssen in The American Journal of Sports Medicine
