# Supplementary material for: Length Changes of the Medial Patellofemoral Ligament During In Vivo Knee Motion: An Evaluation Using Dynamic Computed Tomography
Source: Am J Sports Med. 2023 Nov 13;51(14):3724–31. doi: 10.1177/03635465231205597 (PMC10691293; doi:10.1177/03635465231205597)
Supplement: sj-pdf-1-ajs-10.1177_03635465231205597 – Supplemental material for Length Changes of the Medial Patellofemoral Ligament During In Vivo Knee Motion: An Evaluation Using Dynamic Computed Tomography [file sj-pdf-1-ajs-10.1177_03635465231205597.pdf]

Appendix

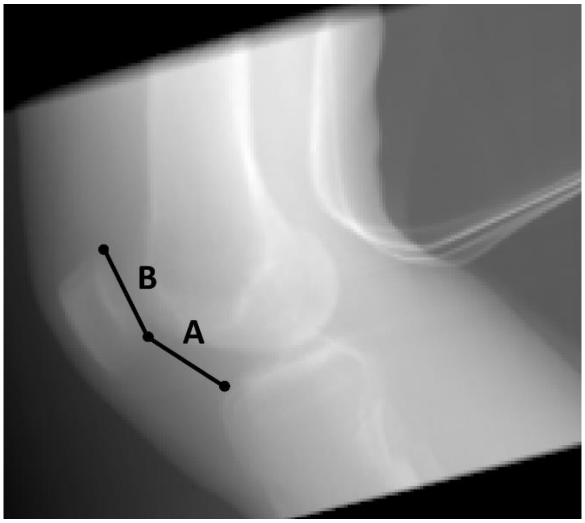

Figure A1. Patellar height assessment using the Caton–Deschamps index (CDI). To assess the patellar height, first, lateral radiographs were generated of the dynamic CT scans when knees were in 30° flexion. An experienced orthopedic surgeon then selected three points representing the CDI (black dots). Subsequently, the CDI was calculated as A/B. A CDI <0.6 was classified as patella baja, a 0.6 ≤ CDI ≤ 1.2 as normal and a CDI >1.2 as patella alta.

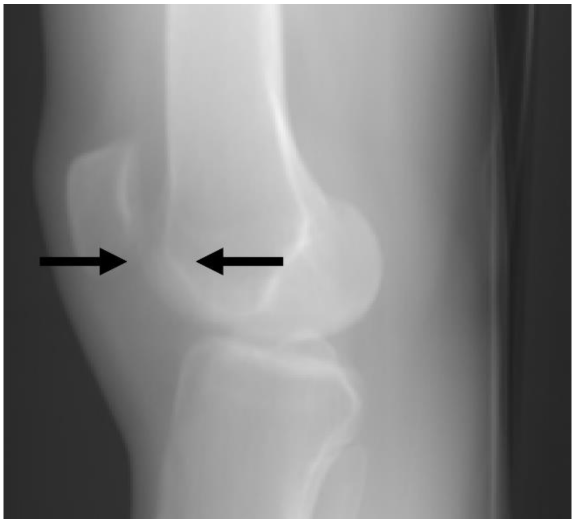

Figure A2. Assessment of trochlear dysplasia according to the criteria of Dejour et al.[8] To assess trochlear dysplasia, lateral radiographs were generated of the static CT scans. An experienced orthopedic surgeon evaluated the presence of trochlear dysplasia in these projections. Trochlear dysplasia was confirmed with CT scans.

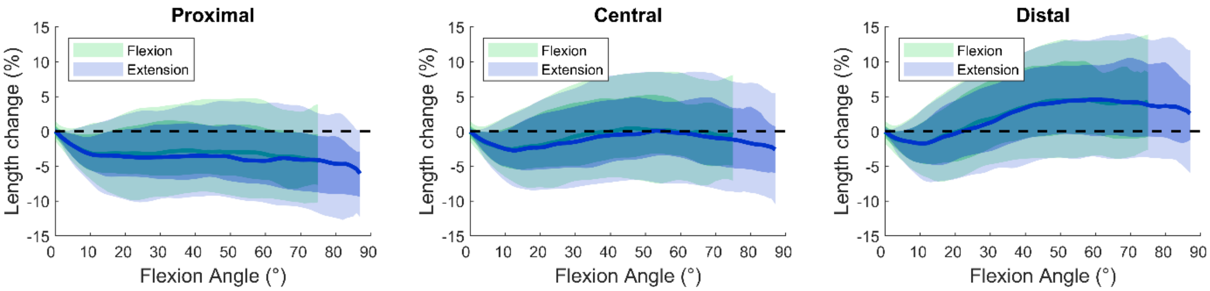

Figure A3. Percentual MPFL length changes relative to full extension for the proximal, central, and distal patellar attachment. The green line and shadings represent MPFL length changes between 0 and 75° of knee flexion. The blue line and shadings represent mirrored MPFL length changes between 0 and 90° of knee extension. Solid lines represent inter-subject medians, dark shadings represent ± IQR (25 – 75%), light shadings represent ± 1.5 IQR (12.5 – 87.5%). The horizontal dotted line represents an isometric

behavior of the MPFL. The self-paced flexion speed of the flexion-extension-flexion movement of most participants was too slow. As a result, only a small minority of the participants reached a knee angle of 90° within the scanning time in the flexion phase. All flexion and extension angles represented in this figure are reached by ≥35% of the participants.

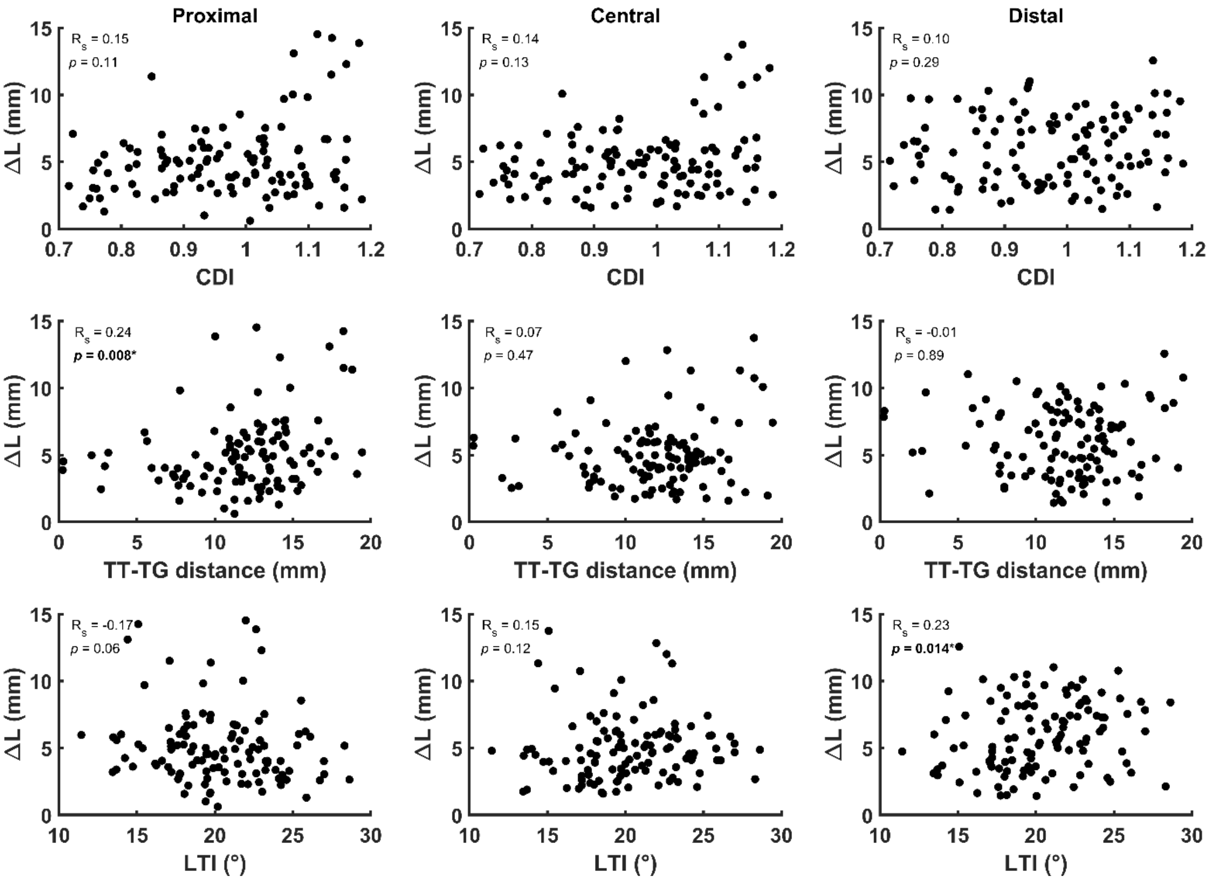

Figure A4. Scatterplots showing the correlation between the absolute overall MPFL length changes between 0 and 90° of knee flexion (ΔL) and anatomical parameters of the patellofemoral joint proximal, central, and distal patellar attachment. Each dots represents the overall MPFL length change in one knee, n = 115 per plot. The plots include Spearman's correlation coefficients (Rs) and statistical significance (P) (see also Table 2). Significant P values are indicated in bold format and marked with an asterisk (*). CDI, Caton-Deschamps index; TT-TG, tibial tuberosity–trochlear groove; LTI, lateral trochlear inclination.
